# Supplementary material for: Low-Molecular Pyrazine-Based DNA Binders: Physicochemical and Antimicrobial Properties
Source: Molecules. 2022 Jun 9;27(12):3704. doi: 10.3390/molecules27123704 (PMC9228100; doi:10.3390/molecules27123704)
Supplement: Supplementary file 1 [file molecules-27-03704-s001.zip › molecules-1713435-supplementary.pdf]

Supplementary Material

Low-molecular pyrazine-based DNA binders: physicochemical and antimicrobial properties

Paulina Mech-Warda[1], Artur Giełdoń[1], Anna Kawiak[2], Natalia Maciejewska[3], Mateusz Olszewski[3], Mariusz Makowski[1], Agnieszka Chylewska[1,*]

[1]*University of Gdańsk, Faculty of Chemistry, Department of Bioinorganic Chemistry, ul. Wita Stwosza 63, 80-308 Gdańsk, Poland*

[2]*Intercollegiate Faculty of Biotechnology of University of Gdańsk and Medical University of Gdańsk, Institute of Biotechnology, ul. Abrahama 58, 80-307 Gdańsk, Poland*

[3]*Faculty of Chemistry, Gdańsk University of Technology, Gabriela Narutowicza 11/12, 80-233, Gdańsk, Poland*

Paulina Mech-Warda
ORCID: 0000-0003-1176-6688
mail: paulina.mech@phdstud.ug.edu.pl

Artur Giełdoń
ORCID: 0000-0003-0415-9214
mail: artur.gieldon@ug.edu.pl

Anna Kawiak
ORCID: 0000-0001-8105-2555
mail: anna.kawiak@biotech.ug.edu.pl

Natalia Maciejewska
ORCID: 0000-0001-9942-285X
mail: natalia.maciejewska@pg.edu.pl

Mateusz Olszewski
ORCID: 0000-0002-1952-4985
mail: mateusz.olszewski@pg.edu.pl

Mariusz Makowski
ORCID: 0000-0002-7342-722X
mail: mariusz.makowski@ug.edu.pl

Agnieszka Chylewska (*corresponding Author*)
ORCID: 0000-0001-7413-1503
mail: agnieszka.chylewska@ug.edu.pl

Structure

Table S1. Selected bond lengths, angles and dihedral angles in the optimized structure of 2-chloro-3-hydrazinopyrazine. Atom numbering is shown in **Figure 2** (the main text).

Atoms	Bond length [Å]	Atoms	Angle [°]	Atoms	Dihedral angle [°]
N1-C2	1.298	N1-C2-C3	122.93	N4-C3-N7-N8	-7.01
C2-C3	1.426	C2-C3-N4	119.03	N4-C3-N7-H12	-169.52
C3-N4	1.332	C3-N4-C5	117.72	C3-N7-N8-N13	70.45
N4-C5	1.340	N4-C5-C6	122.33	C3-N7-N8-H14	-45.27
C5-C6	1.383	C5-C6-N1	120.27	N7-C3-C2-Cl9	-1.33
C6-N1	1.348	C6-N1-C2	117.71		
C2-Cl9	1.767	N1-C2-Cl9	118.01		
C3-N7	1.365	N4-C3-N7	118.92		
N7-N8	1.403	C3-N7-N8	123.06		
C5-H10	1.085	C3-N7-H12	119.37		
C6-H11	1.083	N7-N8-H13	108.59		
N7-H12	1.007	N7-N8-H14	109.04		
N8-H13	1.017				
N8-H14	1.018				

Gibbs Free Energy of the compound -834.667217 Hartree/part.

Vibrational spectra

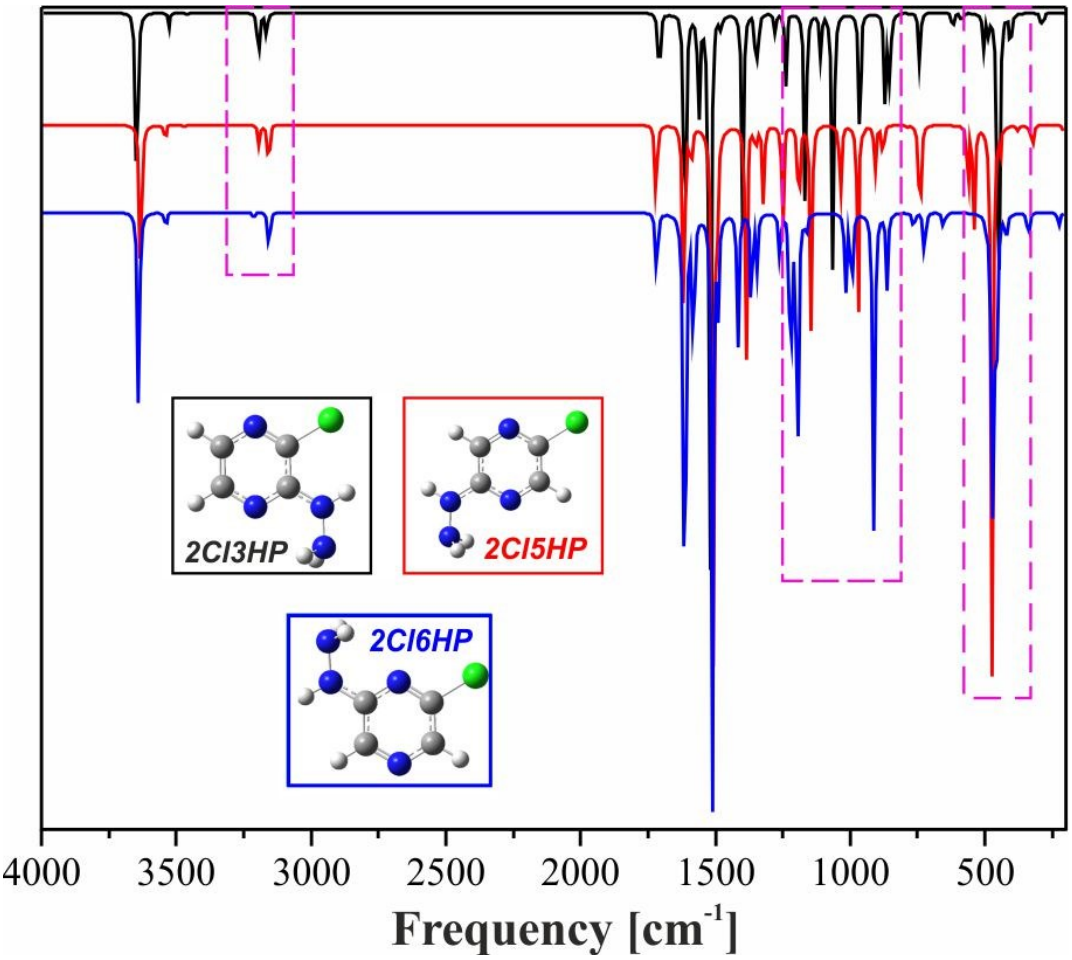

Figure S1. IR spectra computed at B3LYP/6-311+G** level of theory for 2-chloro-3-hydrazinepyrazine (black), 2-chloro-5-hydrazinepyrazine (red), and 2-chloro-6-hydrazinepyrazine (blue) with corresponding structures in frames.

Frontier molecular orbitals (FMOs)

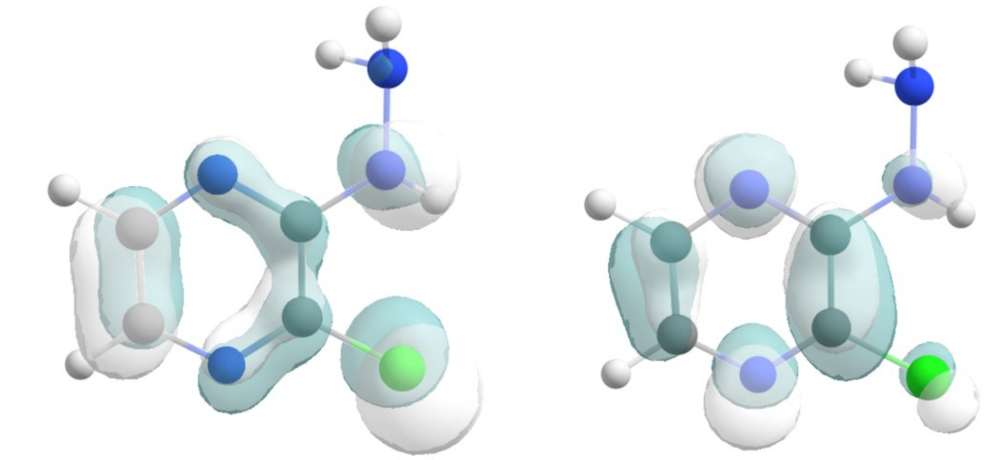

Figure S2. HOMO and LUMO distribution of 2Cl5HP calculated at B3LYP/G-311+G** *in vacuo*. Calculated energy values are as follows: HOMO: -7.096513164; LUMO: -4.11439392 and HOMO-LUMO gap: 2.98212 [eV].

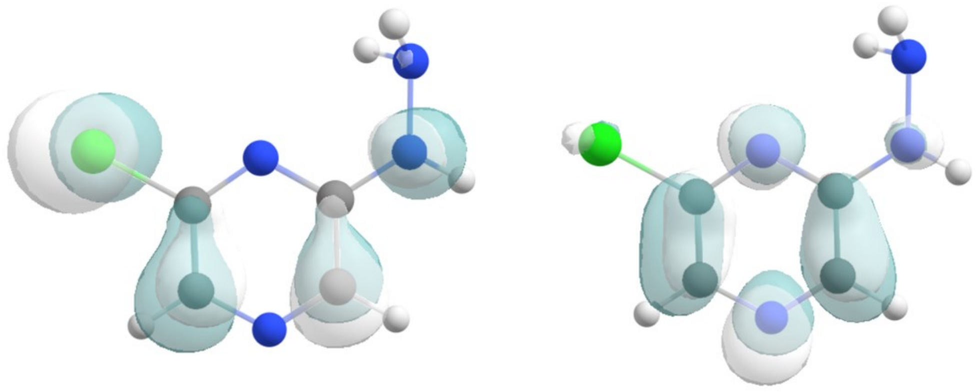

Figure S3. HOMO and LUMO distribution of 2Cl6HP calculated at B3LYP/G-311+G** *in vacuo*. Calculated energy values are as follows: HOMO: -7.230394236; LUMO: -4.2398394 and HOMO-LUMO gap: 2.99056 [eV].

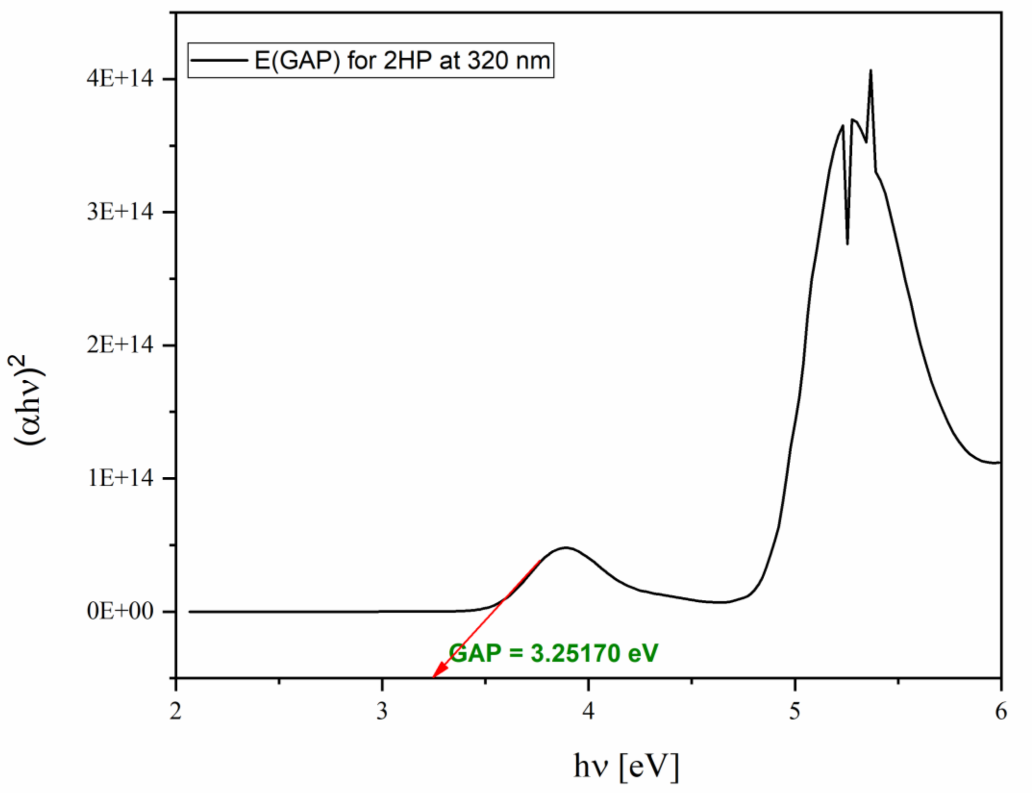

Figure S4. HOMO-LUMO gap: 3.25170 [eV] obtained for 2HP calculated by the experimental spectrum. The GAP value is in good agreement with this calculated at B3LYP/G-311+G** *in vacuo*.

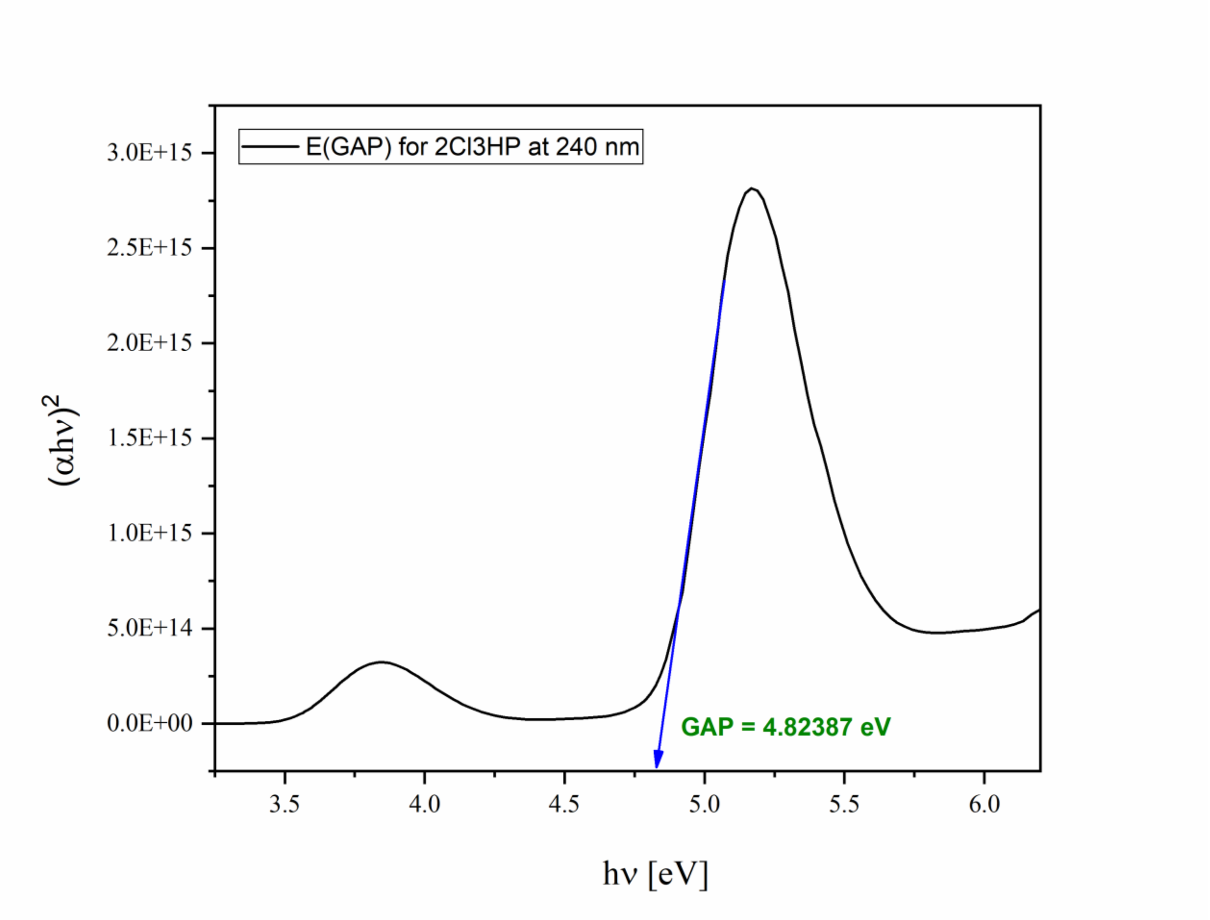

Figure S5. HOMO-LUMO gap: 4.82387 [eV] obtained for 2Cl3HP calculated by the experimental spectrum. The GAP value is in good agreement with this calculated at B3LYP/G-311+G** *in vacuo.*

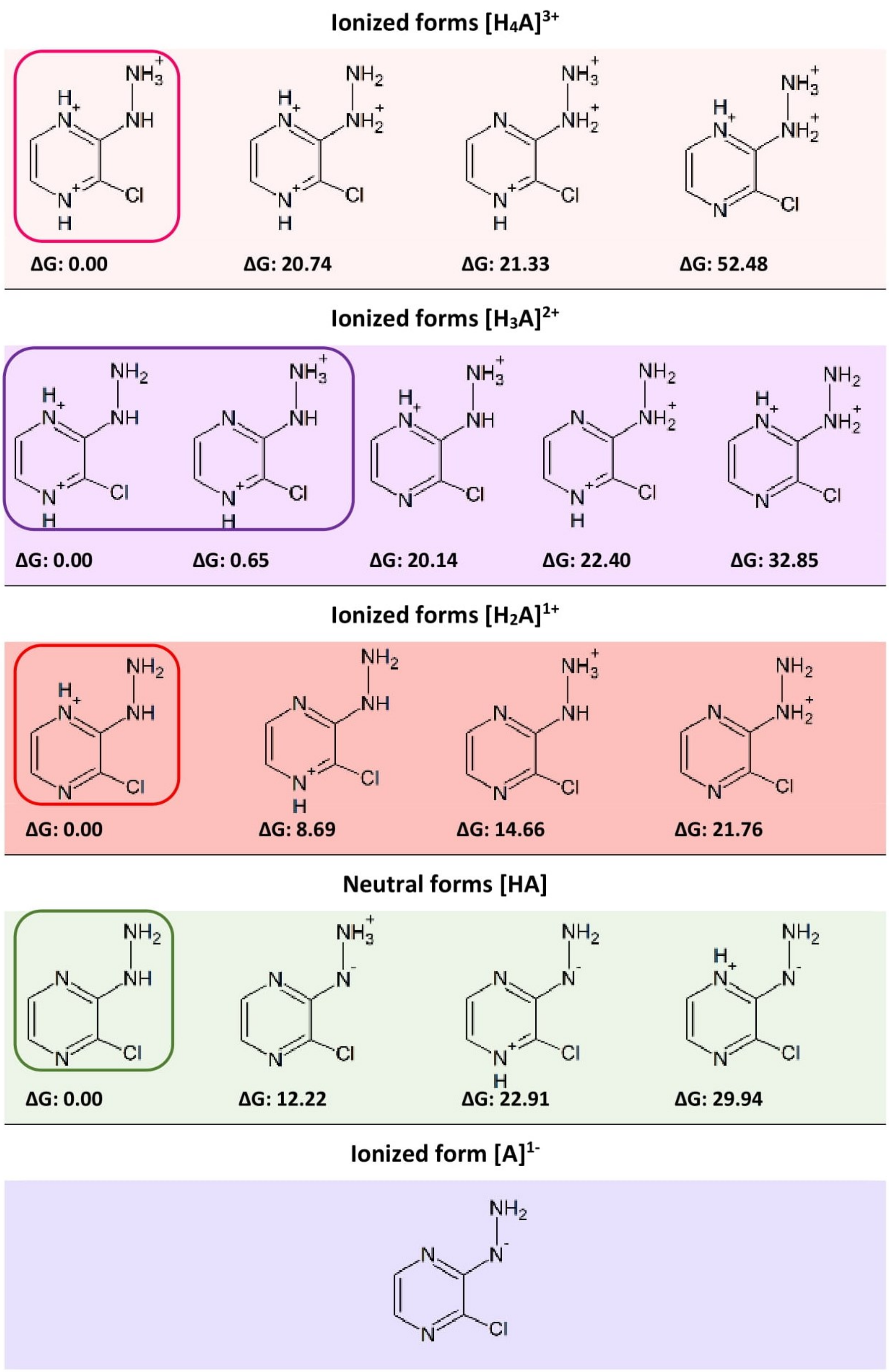

Figure S6. Proposed ionic forms present in the solvent (water) together with the relative Gibbs free energies (ΔG) in kcal/mol for each protonation site and neutral (zwitterion) form.

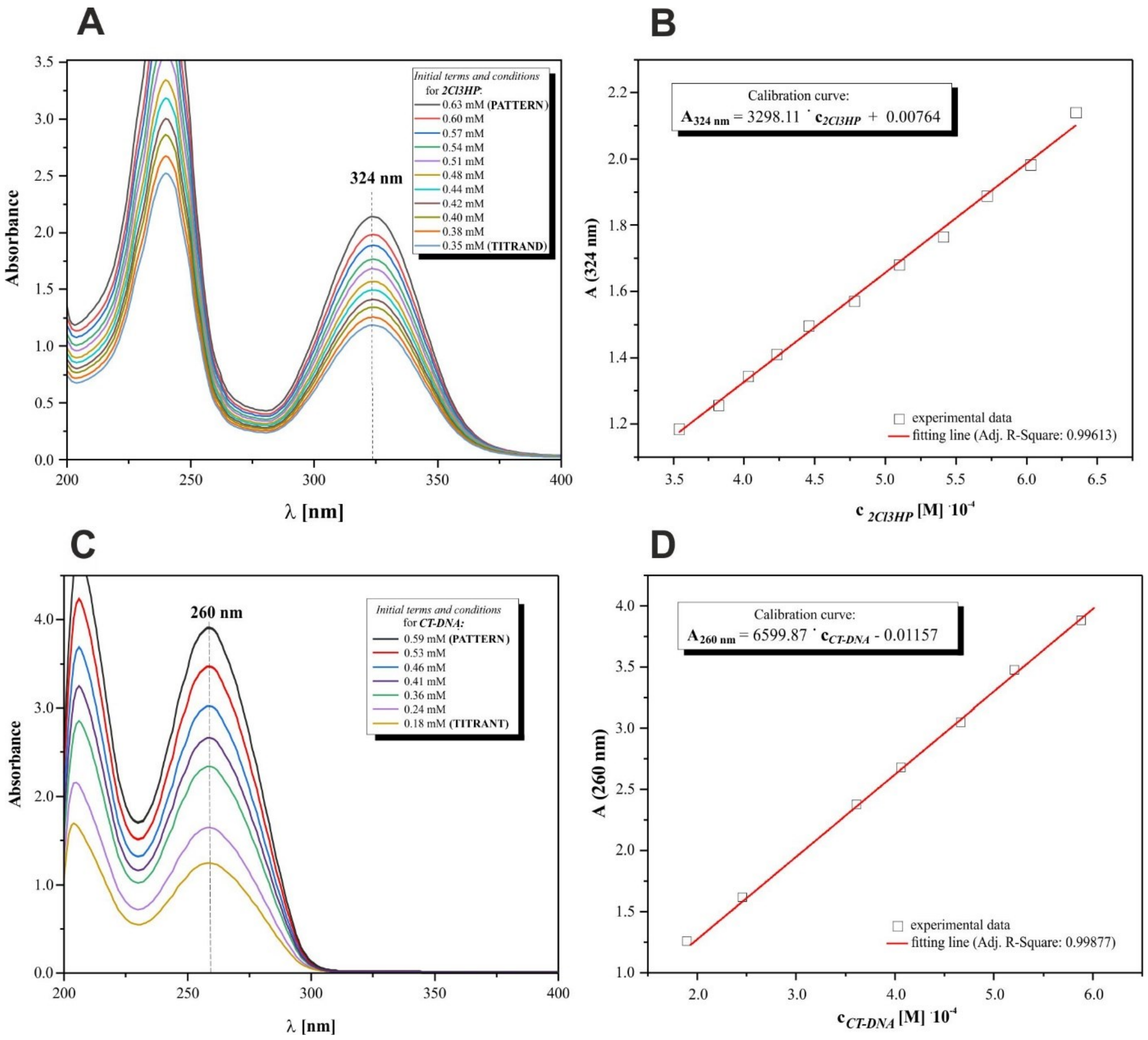

Figure S7. Calibrations of the samples studied: **A.** UV spectra of different concentrations of *2Cl3HP* solutions (0.63÷0.35 mM); **B.** linearity obtained for *2Cl3HP* tested; **C.** electronic spectra of different DNA solution concentrations (0.59÷0.18 mM), **D.** linearity obtained for *CT*-DNA tested.
